# Clinicopathological Characteristics of Plurihormonal Pituitary Adenoma

**DOI:** 10.3389/fsurg.2022.826720

**Published:** 2022-02-25

**Authors:** Ruoyu Shi, Xueyan Wan, Zisheng Yan, Zhoubin Tan, Xiaojin Liu, Ting Lei

**Affiliations:** Department of Neurosurgery, Tongji Hospital, Tongji Medical College, Huazhong University of Science and Technology, Wuhan, China

**Keywords:** plurihormonal pituitary adenoma, clinicopathological characteristics diagnosis, personalized therapy, cell culture, SOX2

## Abstract

**Background:**

As the pathogenesis of plurihormonal pituitary adenoma (PPA) is unclear and the diagnostic criteria are inconsistent, clinicians still find it challenging to diagnose. To analyze the relationship between clinical and pathological characteristics in PPA.

**Methods:**

The clinical data of patients with 70 PPAs admitted during 2008–2010 and 2019–2020 were collected and analyzed. In particular, hormone examination using cell culture supernatant was performed to confirm PPA cases from 2019 to 2020.

**Results:**

PPA accounted for 13% of all pituitary cases recorded in the same period. There were 30 men and 40 women. Fifty-three percent of patients had one endocrine manifestation, and 1% presented with two endocrine symptoms. However, none of the patients had three endocrine manifestations. The level of one and two types of hormones was elevated in 52 (74.3%) and 5 (7.1%) patients, respectively and that of three types of hormones was increased only in one patient. Immunohistochemical staining for PRL + TSH or FSH/LH was most commonly performed (*n* = 17), followed by that for PRL + GH + ACTH and PRL + GH + TSH or FSH/LH (*n* = 14) and PRL + ACTH (*n* = 10). The primary culture results *in vitro* were consistent with the pathological findings in five (41.7%) patients. Moreover, 4 of 12 patients diagnosed with PPA during 2019–2020 tested positive for SOX2.

**Conclusion:**

The pathogenesis of PPA remains elusive due to the lack of specific clinical symptoms and endocrine changes. Examination of hormones on tumor culture supernatant is helpful for its diagnosis.

## Introduction

Plurihormonal pituitary adenoma (PPA) is a type of pituitary adenoma that expresses two or more types of pituitary adenoma hormones in addition to growth hormone/prolactin or follicle-stimulating hormone β subunit/luteinizing hormone β-subunit (1). PPA expresses multiple hormones. However, clinically, it is commonly characterized by the expression of one type of hormone, or patients are asymptomatic. Due to the use of poor diagnostic techniques, PPA is often misdiagnosed in the past. With the development of technologies such as electron microscopy, immunoelectron microscopy, and immunohistochemistry, as well as a better understanding of this tumor, the proportion of patients diagnosed with PPA has been increasing.

As the pathogenesis of PPA is unclear and the diagnostic criteria are inconsistent, clinicians still find it challenging to diagnose. At present, most PPAs are diagnosed based on the following aspects: (1) clinical symptoms and endocrine activity, (2) imaging and intraoperative findings, (3) histology, (4) immunohistochemistry, and (5) ultrastructure (2). However, due to limitations in testing personnel and economic conditions, the wide use of the diagnostic standard, particularly ultrastructure testing, is challenging to implement in clinical practice. Therefore, there are still challenges such as perfecting the diagnostic criteria for PPA and simplifying the diagnostic process, and these should be addressed urgently. The current study aimed to analyze the relationship among clinical manifestations, serological hormone levels, and immunohistochemical results in patients with PPA. Moreover, the clinicopathological characteristics of PPA were analyzed, and the diagnostic criteria for PPA were improved.

## Patients and Methods

The clinical data of patients with PPA admitted to our department from 2008 to 2010 were retrospectively analyzed. Moreover, the clinical, imaging, and laboratory examination data were collected. The inclusion criteria were as follows: (1) patients who underwent complete pituitary magnetic resonance imaging and serum endocrinology examination before surgery and (2) those who had pathological and immunohistochemical examination of tumor tissue, sellar dura, and sphenoid sinus mucosa samples collected during surgery and who have a detailed pathological report. In addition, the imaging and laboratory examination data of patients diagnosed with pituitary adenoma from 2019 to 2020 were prospectively analyzed. Pit-1, SF-1, or T-pit were immunohistochemical stained since 2020 and only three patients had the result of transcription factors. The primary culture of tumor tissues was performed to detect the secretion of tumor cells.

### Primary Culture and Hormone Detection

On the first day, the fresh pituitary tumor tissue samples collected during surgery were soaked in 2 mL DMEM medium containing 10% fetal bovine serum, 100 mg/L streptomycin, 105 U/L penicillin, 10 mmol/L HEPES, and essential amino acids. Then, they were quickly transported to the laboratory and were placed in a biological safety cabinet. Ophthalmological scissors was used to cut the tumor specimens into small tissue sections measuring <1 mm^3^ in a 35-mm diameter petri dish. Then, they were rinsed with PBS and centrifuged three times at 1,000 r/min each for 5 min. The supernatant was discarded. Then, 2 mL DMEM medium was used. The tumor tissue was cultured in an incubator at a constant temperature of 37°C for 24 h. Next, the supernatant was discarded and replaced with 2 mL DMEM medium. The tumor was cultivated in an incubator at a constant temperature incubator of 37°C. Then, the supernatant was collected after culturing for another 24 h. The supernatant culture solution was placed into 15-mL centrifuge tubes, and the presence of TSH and ACTH in the culture medium was evaluated with the Roche electrochemiluminescence method. Next, the Beckman DXI chemiluminescence method was utilized to detect GH, PRL, FSH, and LH in the tumor culture medium.

### Statistical Analysis

All statistical analyses were conducted with the open-source statistical package R version 4.0.4. Continuous variables were presented as mean ± standard deviation.

## Results

### Clinical Data

In total, 535 patients with pituitary adenoma were treated. Among them, 70 had PPA (58 diagnosed in 2008–2010 and 12 in 2019–2020). The clinical data of 70 patients (30 men and 40 women) were collected. The follow-up period ranged from 3 to 156 months, and patients were aged 13–70 (average: 41) years.

### Immunohistochemistry Result and Its Relationship With Tumor Size and Aggressiveness

Histochemical staining for PRL + GH + ACTH was most commonly performed, and this method was used to diagnose 14 patients (*n* = 3, microadenoma; *n* = 5, large adenoma; *n* = 6, unknown; and *n* = 2, intraoperative and postoperative diagnoses of invasive pituitary tumor). In total, 14 patients tested positive for PRL + GH + TSH or FSH or LH or FSH/LH. Among them, one presented with microadenoma and 12 with a large adenoma. However, the type of tumor was unknown in one patient, and eight patients presented with invasive pituitary tumor after surgery. Moreover, 17 patients tested positive for PRL + TSH or FSH or LH or FSH/LH. Among them, two presented with microadenomas and eight with a large adenomas. Then, the type of tumor was unknown in seven cases, and seven patients were diagnosed with invasive pituitary tumors after surgery. Table 1 shows the immunohistochemical results and tumor size and invasiveness.

**Table 1:** The combination of immunohistochemistry and its relationship with tumor size and aggressiveness.

**Immunochemistry**	***n***	**Size**			**Aggressive**
		**Micro**	**Large**	**Unclear**	
PRL + GH + ACTH	14	3	5	6	2
PRL + GH + TSH or Gn	14	1	12	1	8
PRL + ACTH	10	0	8	2	6
PRL + TSH or Gn	17	2	8	7	7
PRL + TSH + LH	1	0	1		0
PRL + TSH + ACTH	1		1		1
GH + ACTH	3	1	2		0
GH + TSH	1	0	1		1
PRL + GH + ACTH + Gn or TSH	7	4	2	1	3
GH + Gn	1		1		1
All positive	1		1		1
Total	70	11	42	17	30

*Gn means FSH or LH or FSH/LH*.

### Relationship Among Immunohistochemistry Results, Clinical Hormone Levels, and Endocrine Symptoms

In total, 40, 11, 10, 8, and 4 patients had high serum PRL, GH, ACTH, TSH, and GH and PRL levels, respectively. Moreover, one had elevated GH and ACTH levels, and only one patient presented with high PRL, TSH, and ACTH levels (Table 2). The positivity rate of PRL immunohistochemistry was the highest, accounting for 91% of all cases. Only 57 had high serum hormone levels, and only 24% presented with endocrine symptoms. The positivity rate of GH immunohistochemistry was 60%. Only 16% of patients had high serum hormone levels. Moreover, 16% had endocrine symptoms. The positivity rate of ACTH immunohistochemistry was 51%. Only 14 had high serum hormone levels, and 11% had endocrine symptoms. Table 3 shows the relationship among immunohistochemistry positivity rates, serum hormones levels, and endocrine symptoms.

**Table 2:** Manifestation.

**Manifestation**	***n***
Irregular menstruation, lactation	17
Visual impairment	26
Headache	12
Acromegaly	11
Cushing syndrome	8
Hyperthyroidism	2
others	6

*Others are including dizziness, fatigue, polydipsia and polyuria, weight loss*.

**Table 3:** Relationship among immunohistochemistry, clinical hormone levels, and endocrine symptoms.

**Hormone**	**Immunohistochemistry**	**Elevated hormone levels**	**Endocrine symptoms**
PRL	64	40	17
GH	42	11	11
ACTH	36	10	8
FSH/LH	27	8	0
TSH	19	8	2

### Endocrine Test Using Primary Culture Medium and Immunohistochemistry of Tumor Tissues

In 5 of 12 patients, the endocrine evaluation findings were consistent with the pathological examination results. However, these two examinations obtained conflicting data. That is, the tumor was a silent type of PPA. Table 4 shows the endocrine examination findings in 12 patients.

**Table 4:** Comparison of endocrine test in primary culture medium and immunohistochemical test of tumor tissue.

**Immunohistochemistry**	***n***	**Endocrine test in primary culture medium**					
		**PRL**	**TSH**	**GH**	**FSH**	**LH**	**ACTH**
PRL + TSH	1		1				
ACTH + FSH	1				1		1
PRL + ACTH	2	1					2
GH + PRL + LH	3	3	1	3	1	1	1
GH+PRL + TSH	2	2	2	2			
PRL + ACTH + TSH	1		1				1
GH + PRL + ACTH + FSH + LH + TSH	1	1	1	1	1		1
GH + PRL + ACTH + TSH	1	1	1	1			1

### SOX2 Positivity Rate in Multihormonal Adenoma

Of 12 patients with PPAs, two tested positive for SOX2. Then, one was partially positive, one was 1% positive, and four patients were positive. The positivity rate was 33%.

## Discussion

The diagnosis of PPAs based on clinical symptoms, serum hormone levels, and HE staining results remains debatable (3). Some scholars recommend that all pituitary hormone components in tumor cells should be identified using immunohistochemical methods (3, 4). However, the hormone components detected by immunohistochemistry don't cause elevation of the corresponding serum hormones and produce corresponding endocrine symptoms. For example, in this study (as shown in Table 3), 36 patients tested positive for ACTH and 19 for TSH. However, only 10 patients had high ACTH levels, and 8 had elevated serum TSH levels. Further, only eight and two patients presented with Cushing's syndrome and hyperthyroidism, respectively. The possible reason is that the hormone secreted by the tumor is not biologically active, or it has lost activity after entering the blood circulation (3). In addition, three patients underwent multiple surgeries due to relapse, and the immunohistochemistry results were inconsistent. Thus, the efficacy of immunohistochemistry alone in diagnosing PPA is still debated.

The latest classification of pituitary tumors is based on transcription factors and differentiation drivers in the differentiation pathway of pituitary cells. However, they cannot completely reflect the different endocrine disorders in PPA. Currently, the diagnosis of PPAs is still controversial, and the use of serum hormone levels, clinical manifestations, and pathological results alone is not sufficient. Moreover, few types of multihormonal adenomas are difficult to diagnose (3, 5). Hormonal examination using a tumor cell supernatant cultured *in vitro* is a good diagnostic method. Russel et al. succeeded in culturing pituitary adenoma cells *in vitro* for the first time in 1959, and several studies have assessed the primary culture of pituitary adenoma cells. Adams et al. (6) attempted to culture pituitary tumor tissues *in vitro* to detect hormone secretion. Results showed that this method was convenient and quick, and whole tumor tissues could be used, which has a unique role in diagnosing pituitary adenoma. Some subsequent studies (7–9) reported that non-functional adenomas can secrete LH, FSH, and PRL in cell culture *in vitro*. The *in vitro* culture experiment of functional pituitary adenoma (10–13) has shown that functional pituitary adenoma has a good secretory function *in vitro*. Moreover, previous studies (14, 15) have revealed that the hormones secreted by pituitary adenomas *in vitro* were often different from those observed in immunohistochemistry and serology. Therefore, primary tumor culture *in vitro* is important in assessing the etiology, pathogenesis, diagnosis, and treatment of tumors.

In this study, the tissue samples of 12 patients with PPA were cultured *in vitro*. In most cases, the secretion of multiple hormones could be evaluated. Results showed that only four patients had a single hormone secretion, and the corresponding clinical manifestations were observed. In one case, all pituitary hormones were detected, and some normal pituitary tissues were mixed in the tumor tissue. In 5 of 11 patients, the immunohistochemistry results of six hormones were consistent with *in vitro* culture findings. However, in six patients, the immunohistochemistry results of six hormones were in contrast with *in vitro* culture results. This may be caused by the following: (1) The hormone cell level is low (16). (2) Tumor cells synthesize hormones. However, they are not secreted outside of the cell, or they degrade immediately after secretion (17). (3) Hormones only have immunological activity, not biological (16, 18). Moreover, in three patients, the endocrinologic examination results of the primary tumor culture were in accordance with the serological result. This could be attributed to the following: (1) The cell count is low and is not enough to identify significant serum changes and clinical symptoms (16). (2) After the tumor compresses the normal pituitary gland, pituitary gland atrophy and hormone levels decrease. Meanwhile, the tumor cells produce low hormone levels. The two can cancel each other out, resulting in a relatively normal serum hormone level and absence of clinical manifestations indicating excessive hormone secretion. (3) Patients may have abnormal receptors for this hormone (19). (4) The secreted hormones have been inactivated in the circulation. Tumor culture conditions *in vitro* are relatively stable, which can give full play to the function of the whole tissue. In addition, the hormone secretion time is long, easy to accumulate, and the concentration is high. It has certain advantages in diagnosing the endocrine function of tumors. Hence, it has certain advantages and can be used along with other diagnostic methods. However, more specific and effective culture conditions must be explored.

Regarding the pathological assessment of PPA, some scholars (18) believe that it is transformed from certain cells during the normal early development of the pituitary gland. In addition, previous studies (20, 21) have shown that it is transformed from stem cells. In this study, the SOX2 stem cell markers were stained in 12 patients diagnosed with PPA from 2019 to 2020. Only four patients tested positive, and the positivity rate is of 33%. The pathogenesis of PPA could not be completely explained by the origin theory of stem cells. Therefore, the pathogenesis and tumor origin of PPA must be further assessed.

## Conclusion

PPA is commonly characterized by a large adenoma with an aggressive biological behavior. The immunohistochemistry of tumor tissues in patients with PPA shows positivity to multiple hormones. However, it does manifest as increased corresponding hormones in serum and clinical symptoms, but manifested as endocrine symptoms of only one increased serum hormone or non-functioning state. PPA is often diagnosed based on clinical manifestations, serum hormone levels, tumor immunohistochemical staining and hormone evaluation (using a supernatant for tumor cells cultured *in vitro*).

## Data Availability Statement

The original contributions presented in the study are included in the article/Supplementary Material, further inquiries can be directed to the corresponding author.

## Ethics Statement

The studies involving human participants were reviewed and approved by Medical Ethics Committee of Tongji Hospital, Tongji Medical College, Huazhong University of Science and Technology. The patients/participants provided their written informed consent to participate in this study.

## Author Contributions

All authors made a substantial contribution to all aspects of the preparation of this manuscript.

## Conflict of Interest

The authors declare that the research was conducted in the absence of any commercial or financial relationships that could be construed as a potential conflict of interest.

## Publisher's Note

All claims expressed in this article are solely those of the authors and do not necessarily represent those of their affiliated organizations, or those of the publisher, the editors and the reviewers. Any product that may be evaluated in this article, or claim that may be made by its manufacturer, is not guaranteed or endorsed by the publisher.
